# Applications and mechanisms of therapeutic ultrasound in skin repair and regeneration

**DOI:** 10.3389/fphys.2026.1981431

**Published:** 2026-10-27

**Authors:** Zeyu He, Shiwen Shao, Yi Fang, Xuegang Xu, Gaosen Zhang

**Affiliations:** 1 Department of Dermatology, First Affiliated Hospital of China Medical University, Shenyang, Liaoning, China; 2 Department of Ultrasound, First Affiliated Hospital of China Medical University, Shenyang, Liaoning, China

**Keywords:** mechanotransduction, skin regeneration, therapeutic ultrasound, transdermal delivery, ultrasound-responsive biomaterials

## Abstract

Ultrasound modulates cellular and tissue processes underlying skin repair and regeneration via thermal and mechanical effects, including cavitation, and can act as an external trigger to facilitate transdermal delivery or activate ultrasound-responsive materials. This review summarizes the acoustic fundamentals and Mechanotransduction pathways of different types of ultrasounds, as well as research advances in wound healing, pathological scar intervention, skin tissue remodeling, skin appendage regeneration, and synergistic applications with ultrasound-responsive materials. Emphasis is placed on the molecular and cellular mechanisms by which ultrasound regulates inflammatory responses, angiogenesis, re-epithelialization, and extracellular matrix remodeling. Currently, apart from studies on focused ultrasound and ultrasound-assisted wound therapy, most supporting evidence is derived from *in vitro* experiments, animal models, or small-sample clinical studies. Inconsistent acoustic parameters and outcome measures, as well as insufficient long-term safety data, represent major barriers to its clinical translation.

## 1 Introduction

The skin is a composite organ that undergoes continuous renewal and dynamic adaptation to external stimuli. In states of chronic inflammation, fibrosis, or aging, impairments such as declined skin barrier function, disordered matrix arrangement, abnormal scar formation, skin laxity, and diminished appendage function may occur. Therefore, regulating skin cell behavior and the tissue microenvironment to promote structural and functional tissue reconstruction is a core focus of skin regeneration research. Here, repair denotes wound closure and restoration of tissue continuity, potentially with scarring; remodeling denotes changes in matrix organization and tissue properties; and regeneration denotes restoration of native-like structure and relevant function, including lost appendages. These processes overlap, but wound closure, collagen deposition, or improved skin contour alone should not be equated with complete skin regeneration.

Therapeutic ultrasound elicits diverse biological effects through thermal and mechanical, effects, with cavitation belonging to the latter. Low-intensity pulsed ultrasound (LIPUS) is associated with cell proliferation and migration, inflammation modulation, angiogenesis, and tissue remodeling. Low-frequency ultrasound temporarily alters skin barrier permeability via cavitation to enhance transdermal drug transport, and its combination with microbubbles further augments local drug delivery. High-intensity focused ultrasound (HIFU) induces local thermal coagulation followed by tissue remodeling, and is used for skin tightening, tissue ablation, and certain scar therapies (Bachu et al., 2021; Haykal et al., 2025; Welc et al., 2025).

At the cellular level, in accordance with general mechanobiological principles, cyclic mechanical stimulation can be converted into biochemical signals via integrin adhesion complexes, the cytoskeleton, and mechanosensitive ion channels. Some *in vitro* studies of LIPUS have observed its effects on mechanosensitive ion channels and activation of related signaling pathways (Kim et al., 2023; Liang et al., 2024). At the tissue level, studies have demonstrated that therapeutic ultrasound modulates inflammation and tissue reconstruction in the skin and its appendages (Boerman et al., 2023; Yu et al., 2024). This review analyzes the associations between acoustic effects of therapeutic ultrasound, cellular perception, signal transduction, and tissue outcomes. It also evaluates evidence regarding wound healing, pathological scars, skin tissue remodeling, appendage regeneration, and ultrasound-responsive biomaterials, with a focus on the value and feasibility of clinical translation of therapeutic ultrasound in the field of skin tissue regeneration.

## 2 Acoustic fundamentals of therapeutic ultrasound

### 2.1 Key acoustic parameters

The biological effects of therapeutic ultrasound are collectively determined by frequency, sound pressure or intensity, pulse parameters, exposure duration, and sound field distribution. Frequency affects wavelength and attenuation of sound waves in tissues; however, the actual penetration depth is also influenced by transducer aperture, focusing mode, tissue acoustic impedance, coupling conditions, and geometric features of the lesion site. Accordingly, therapeutic efficacy cannot be directly compared across studies solely based on frequency or nominal device power (Horne et al., 2020; Majnooni et al., 2022; Padilla and Ter Haar, 2022; Doveri et al., 2024). Parameter heterogeneity can change both physical interactions and biological responses, as illustrated by the contrasting findings in **Table 1** (de Lucas et al., 2021; Vander Horst et al., 2021; Cheng et al., 2023; Jiang et al., 2024; Yang et al., 2026). Following existing reporting recommendations (Padilla and Ter Haar, 2022), studies should specify transducer geometry and field distribution; frequency; calibrated pressure or intensity with spatial and temporal averaging conventions; pulse duration, repetition frequency and duty cycle; session duration, interval and number; coupling, target geometry and measurement conditions; and calibration uncertainty. Temperature and cavitation monitoring should be reported where relevant. Spatial-peak pulse-average, spatial-peak temporal-average and spatial-average temporal-average intensities are not interchangeable. Device power grades or drive voltages should not be treated as calibrated tissue exposure. Dressings and responsive materials may further modify delivered exposure. Standardized reporting, rather than one universal treatment setting, is needed for reproducible comparisons.

**Table 1:** Selected experimental ultrasound exposures models and outcomes.

Study and model	Reported exposure	Biological outcome and interpretation
(Jiang et al., 2024) Fibroblast models including patient-derived keloid fibroblasts; keloid xenografts	110 kHz nominal frequency; selected cell protocol: 300 Vpp, 3 min, 50% duty cycle. System intensity reported as approximately 3 W/cm², without an explicit spatial/temporal averaging label.	Reduced migration and increased apoptosis in keloid-related cells; Piezo1 perturbation supports a functional contribution. This concerns pathological fibroblast suppression, not general promotion of skin regeneration.
(Cheng et al., 2023) Human epidermal keratinocytes	15 min; contact: 1 MHz, 40 or 200 mW/cm²; noncontact: 0.45 MHz, 30 mW/cm². Intensities retained as reported.	Increased migration and calcium-dependent transcription-factor activation; calcium-free medium and channel blockers inhibited responses. Cellular functional evidence; not proof of complete tissue regeneration.
(de Lucas et al., 2021) Human and mouse skin mesenchymal precursors	3 MHz; reported 100 mW/cm²; 20% duty cycle; 1,000 Hz pulse repetition; 20 min/day for 3 days.	No improvement in proliferation or migration under the tested protocol. Functional assays followed cell expansion 5 days after the last exposure. Negative findings are exposure- and model-specific.
(Yang et al., 2026) Diabetic rat wounds with intravenous microbubbles	6.6 MHz; device power grade 5; 100 Hz PRF; 1,200 μs pulse duration; 5 s on/5 s off; 10 min every other day over 18 days.	Improved closure, granulation and angiogenesis with macrophage-marker and inflammatory-signaling changes. Tissue benefit and associations; device power grade is not a calibrated intensity.
(Vander Horst et al., 2021) Full-thickness wounds in diabetic mice	1 MHz; 2 ms pulses; 100 Hz PRF; 0–0.4 MPa; 8 min/day, 5 days/week for 2 or 3 weeks. At 0.4 MPa, reported I_SPTA = 0.7 W/cm².	Increased granulation and re-epithelialization at 0.4 MPa after 2 weeks; dose/time dependence and variable individual responses. Repair outcomes do not demonstrate full skin regeneration.

Selected examples from the existing reference list, not an exhaustive extraction. Device drive voltage, power grade and differently averaged intensities are retained in their original form and must not be treated as equivalent tissue doses. PRF, pulse repetition frequency; Vpp, peak-to-peak drive voltage; I_SPTA, spatial-peak temporal-average intensity.

### 2.2 Mechanical and thermal effects

Mechanical effects include both non-cavitation and cavitation-mediated interactions. Acoustic radiation force transfers acoustic momentum to tissues, while acoustic streaming produces time-averaged fluid motion and shear stress without requiring bubbles. These interactions can load membranes and adhesion structures; their relative contributions depend on exposure and tissue conditions (Ho et al., 2021; Zhang et al., 2021; Padilla and Ter Haar, 2022). The mechanical effects of therapeutic ultrasound act on cell membranes, cell adhesion structures, the cytoskeleton, and the extracellular matrix, thereby modulating cellular mechanosensation and signal transduction. In skin-related research, therapeutic ultrasound induces altered intracellular calcium distribution in keratinocytes and temporarily increases basement membrane permeability (Zheng et al., 2025). Sequential application of low- and high-frequency ultrasound enhances transdermal delivery of macromolecules (Matsubara et al., 2025). These findings indicate that the mechanical actions of therapeutic ultrasound can modify skin barrier status.

Thermal effects arise from local temperature elevation caused by tissue absorption of acoustic energy. The magnitude of temperature rise depends on frequency, sound intensity, duty cycle, exposure time, tissue absorption properties, and local blood flow heat dissipation. HIFU generates thermal coagulation zones via localized energy deposition, which triggers collagen denaturation, immediate contraction, and subsequent tissue remodeling, leading to improvements in skin hydration, elasticity, roughness, and wrinkles (Choi et al., 2024).

Cavitation refers to the oscillation, expansion, or collapse of bubble nuclei in liquids or tissues under alternating sound pressure. Stable cavitation produces bubble-associated microstreaming and shear stress, whereas inertial cavitation may generate more intense microjets, shock waves, and local mechanical damage. Cavitation effects temporarily alter lipid arrangement in the stratum corneum and barrier permeability, thereby promoting transdermal drug transport (Gaikwad et al., 2024). Ultrasound-responsive carriers and phase-change materials can also exploit cavitation to modulate drug delivery into the epidermis and dermis (Zhang Y. et al., 2025) (**Figure 1**). Piezoelectric deformation generates electrical cues, whereas piezocatalysis uses mechanically generated charges to drive interfacial redox reactions (Liu et al., 2023; Li et al., 2025; Zhang Z. et al., 2025). Sonochemical reactions, often associated with cavitation, can contribute to reactive oxygen species generation in suitable systems (Liu et al., 2023; Ma G. et al., 2025). Their contributions require appropriate acoustic, material-only and ultrasound-only controls and should not be assumed to be identical across platforms.

**Figure 1:**
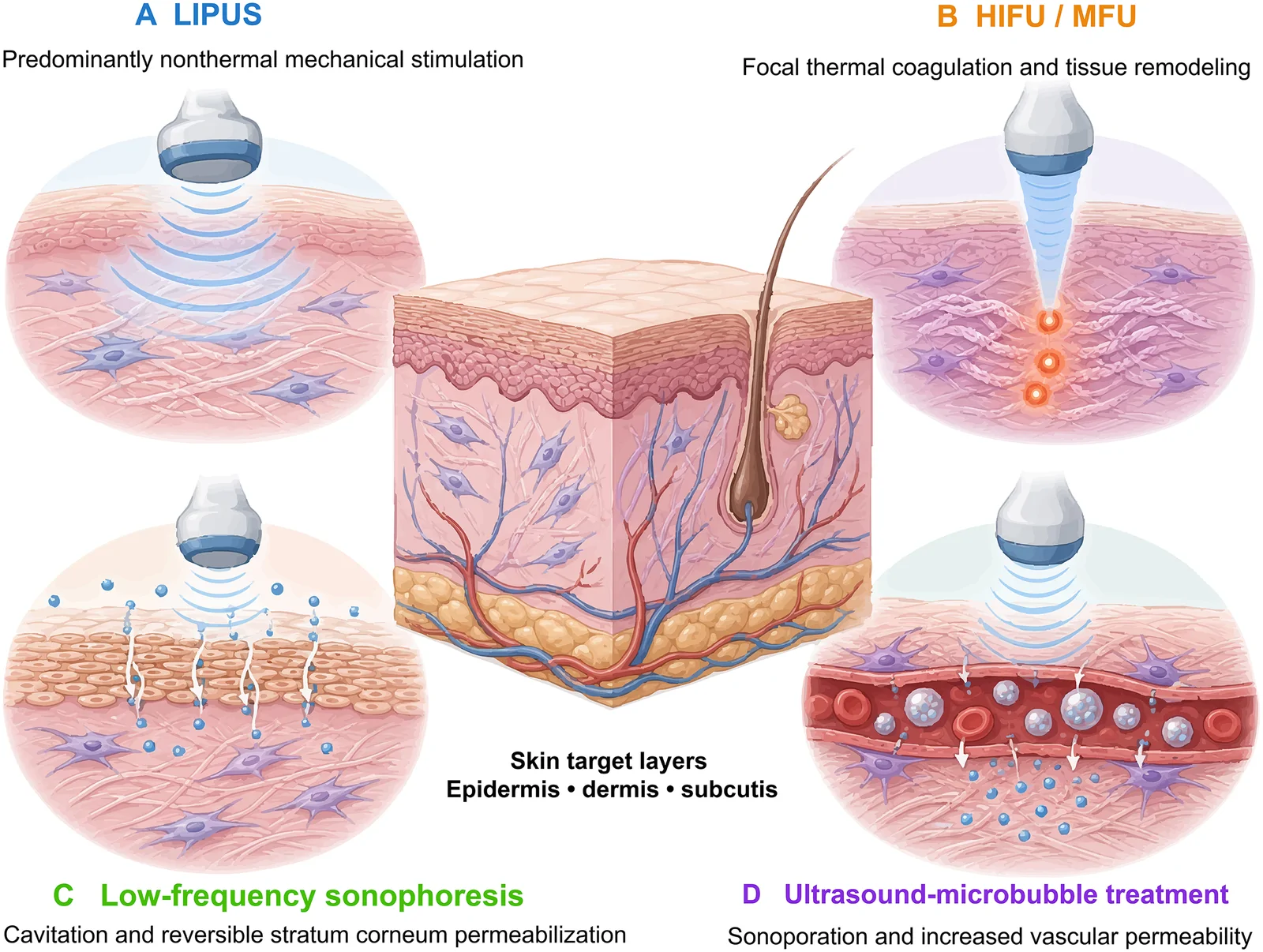
Major ultrasound modalities and their principal tissue targets in the skin. **(A)** Low-intensity pulsed ultrasound (LIPUS) predominantly produces nonthermal mechanical stimulation in the epidermis and dermis. **(B)** High-intensity focused ultrasound (HIFU) and microfocused ultrasound (MFU) generate focal thermal coagulation zones at defined tissue depths, followed by collagen remodeling. **(C)** Low-frequency sonophoresis enhances transdermal transport through cavitation-mediated, reversible permeabilization of the stratum corneum. **(D)** Ultrasound–microbubble treatment induces sonoporation and increases vascular permeability, thereby facilitating localized delivery. The biological effects and target depths vary with ultrasound modality, frequency, acoustic intensity, pulse characteristics, and focusing strategy and should therefore not be considered interchangeable.

## 3 Mechanobiological mechanisms of ultrasound mechanical effects in regulating skin repair and regeneration

### 3.1 Integrin–focal adhesion–cytoskeleton axis

Mechanobiological studies have shown that mechanical forces modulate migration, proliferation, myofibroblast differentiation, and matrix synthesis in skin fibroblasts (Berry et al., 2023). Matrix components such as collagen influence re-epithelialization via integrin signaling (Mistry et al., 2024); matrix composition and stiffness also alter mechanosensation at focal adhesions (Sturgess et al., 2024), and epidermal integrin-mediated secretory signals participate in local microenvironment regulation (Longmate, 2024). These findings establish the biological basis for the integrin–focal adhesion–cytoskeleton axis in sensing mechanical forces.

Following LIPUS stimulation of human skin fibroblasts, intracellular β1 integrin, Rho/ROCK, Src, and ERK1/2 are activated; conversely, blockade of β1 integrin or inhibition of ROCK, Src, and MEK abrogates these effects (Zhou et al., 2004). Studies have also reported altered expression of p38 and ERK1/2 in human dermal fibroblasts after high-intensity ultrasound exposure (Lee et al., 2021). LIPUS-associated cytoskeletal and junctional alterations in keratinocyte and melanoma cell models represent a separate experimental context (Udroiu et al., 2025).

### 3.2 Mechanosensitive ion channels and calcium ions

Piezo1 is one of the most extensively studied mechanosensitive ion channels in therapeutic ultrasound research on skin. Studies using patient-derived keloid fibroblasts have shown that 110 kHz low-frequency ultrasound inhibits fibroblast migration and promotes apoptosis via Piezo1 activation (Zhang Z. et al., 2025). Additionally, low-intensity ultrasound treatment induces redistribution of intracellular calcium in keratinocytes and increases basement membrane permeability (Zheng et al., 2025), and LIPUS modulates migration through calcium-dependent signaling in human. epidermal keratinocytes (Cheng et al., 2023). In contrast, radiation-force-induced Piezo1 activation in osteoblastic cells is non-skin evidence (Zhang et al., 2021). These findings support model-dependent mechanosensitive responses, rather than a universally established regenerative pathway.

### 3.3 Primary cilia and nuclear mechanotransduction

Primary cilia sense fluid shear stress and are involved in multiple developmental signaling pathways. Focused ultrasound regulates cilia and adipogenic responses in adipose-derived stem cells within subcutaneous adipose tissue (Oh et al., 2022).

YAP and TAZ are key transcriptional regulators in the Hippo pathway, whose subcellular localization is influenced by matrix stiffness, cell spreading area, and cytoskeletal tension. Studies in human dermal fibroblasts have linked changes in cell size and mechanical state to YAP/TAZ and CCN2 regulation, which contribute to matrix alterations associated with skin aging (Xiang et al., 2021; Qin et al., 2022). TAZ may also attenuate UVA-induced photoaging in skin fibroblasts (Wang et al., 2023).

Aberrant expression of Hippo pathway components and integrin-linked kinase has been observed in hypertrophic scars and keloids (Petrou et al., 2022), and studies of scar-free wound healing in spiny mice implicate the Hippo-YAP pathway and myofibroblast fate in regenerative skin repair (Brewer et al., 2021). These YAP/TAZ studies provide skin-mechanobiology rationale but do not directly validate YAP/TAZ as a mediator of ultrasound-induced skin regeneration (Brewer et al., 2021; Xiang et al., 2021; Petrou et al., 2022; Qin et al., 2022; Wang et al., 2023) (**Figure 2**).

**Figure 2:**
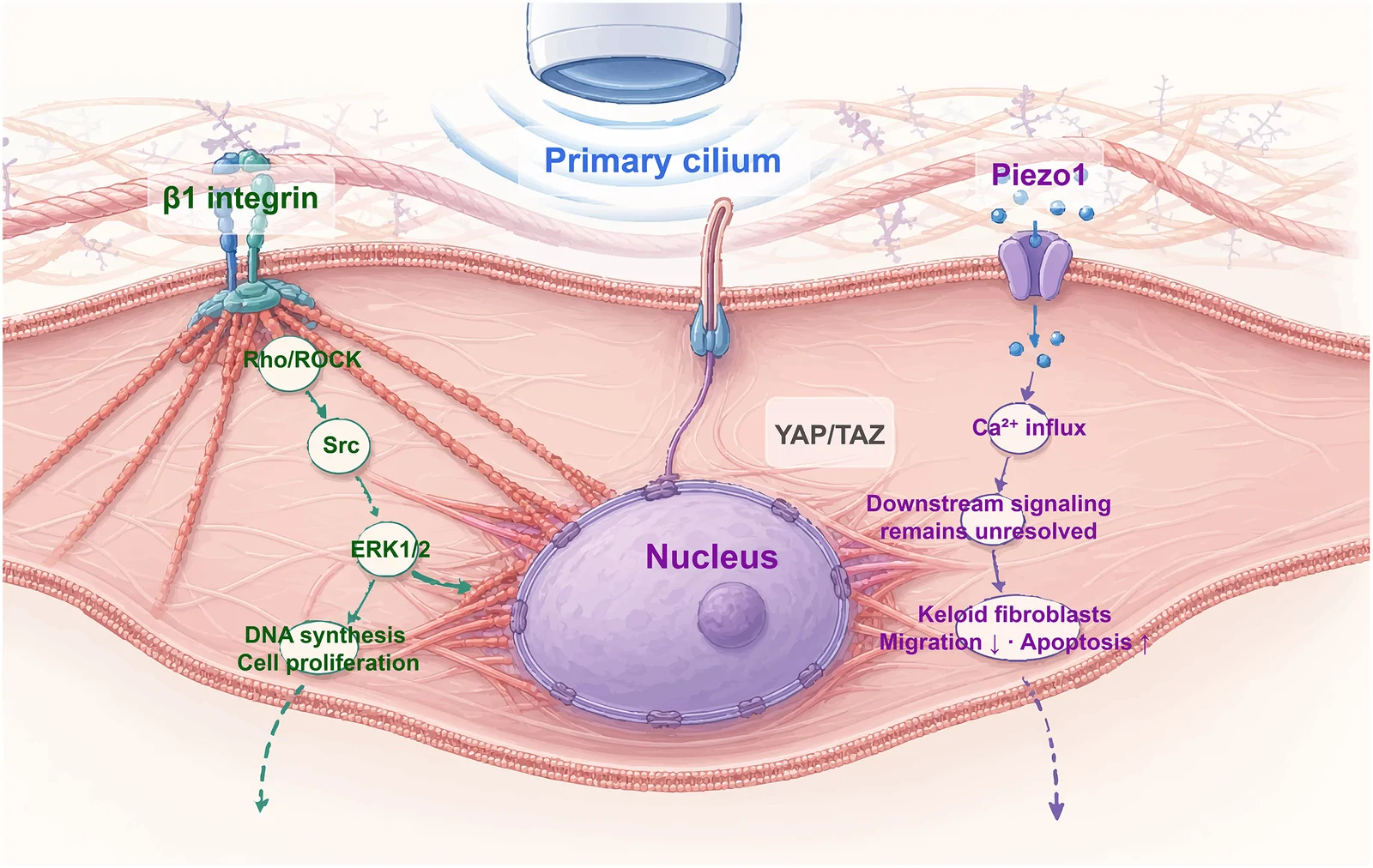
Ultrasound-induced mechanotransduction pathways in skin cells. Ultrasound-generated mechanical stimuli can be sensed by membrane-associated mechanoreceptors and transmitted through the cytoskeleton to intracellular signaling networks and the nucleus. In human skin fibroblasts, the β1 integrin–Rho/ROCK–Src–ERK1/2 axis has been implicated in LIPUS-induced DNA synthesis and cell proliferation, with functional evidence obtained using pathway inhibition. In keloid fibroblasts, low-frequency ultrasound activates Piezo1 and promotes Ca²^+^ influx, accompanied by reduced cell migration and increased apoptosis; however, the downstream signaling events connecting Ca²^+^ influx to these cellular responses remain incompletely defined. Primary cilia and YAP/TAZ may also participate in ultrasound mechanotransduction, although direct evidence in ultrasound-treated skin cells remains limited. Solid arrows indicate direct ultrasound evidence with functional validation, whereas dashed arrows indicate correlative, indirect, or proposed relationships. LIPUS, low-intensity pulsed ultrasound.

## 4 Signaling networks potentially involved in skin repair and regeneration

### 4.1 Pathways related to cell proliferation and migration

The MAPK/ERK and PI3K/Akt pathways are widely involved in cell proliferation, survival, migration, and metabolic regulation. In human skin fibroblasts, LIPUS-induced DNA synthesis depends on β1 integrin and Rho/ROCK/Src/ERK signaling (Zhou et al., 2004). High-intensity ultrasound treatment of human dermal fibroblasts enhances cell migration and induces p38- and ERK1/2-related changes (Lee et al., 2021).

In a rat model of acute skin wounds, ultrasound combined with microbubbles improved re-epithelialization, accompanied by downregulation of miR-26b-5p and altered GSK3β-related signaling (Huang et al., 2025). In contrast, no significant changes in proliferation or migration were observed in mesenchymal precursor cells following therapeutic ultrasound stimulation (de Lucas et al., 2021).

### 4.2 Signaling pathways involved in extracellular matrix remodeling and fibrosis

The TGF-β/Smad pathway regulates fibroblast activation, myofibroblast differentiation, and collagen synthesis. In photoaged mice treated with focused ultrasound, changes in TGF-β and matrix metalloproteinase markers, along with improved collagen formation and arrangement, have been reported (Lee et al., 2022); these changes most likely represent tissue remodeling responses following thermal coagulation. Studies of ultrasound-responsive piezoelectric membranes and conductive hydrogels have demonstrated improved collagen deposition, alignment, and scar outcomes (Yao et al., 2025; Zhang Z. et al., 2025). Changes in TGF-β expression or collagen organization alone do not establish Smad-dependent causation of the ultrasound response.

Rho/ROCK and YAP/TAZ are key regulatory pathways linking cytoskeletal tension to extracellular matrix remodeling. Research has shown that Rho/ROCK is involved in specific LIPUS-induced DNA synthesis in human skin fibroblasts (Zhou et al., 2004), and Piezo1-dependent responses occur when low-frequency ultrasound acts on keloid fibroblasts (Jiang et al., 2024).

### 4.3 Angiogenesis-related signaling pathways

In acute and diabetic skin wound models, respectively, ultrasound combined with microbubbles improves angiogenesis-related markers and wound healing (Huang et al., 2025; Yang et al., 2026). LIPUS also enhances uptake of adipose-derived stem cell exosomes by target cells and augments their pro-angiogenic effects (Zhong et al., 2024). In a rabbit capsule tissue model, LIPUS increased tissue perfusion, vascular density, and expression of VEGF and basic fibroblast growth factor (Yu et al., 2021). In human umbilical vein endothelial cells, LIPUS promotes angiogenesis, accompanied by alterations in the AKT pathway and DNA methylation (Li et al., 2022). In patients with chronic limb-threatening ischemia, LIPUS treatment improved some vascular function and skin perfusion parameters (Kajikawa et al., 2025). The capsule, endothelial-cell and limb-ischemia findings support vascular responses in their respective settings, but do not establish the same causal pathway in ultrasound-treated skin wounds.

### 4.4 Inflammation modulation and immune cell responses

Inflammatory responses are integral to skin regeneration. The immunomodulatory effects of LIPUS are associated with inflammation-related signaling pathways such as NF-κB and MAPK (Liang et al., 2024). In diabetic skin wound models, ultrasound combined with microbubbles shifts macrophage polarization, alleviates persistent pro-inflammatory responses, and promotes wound healing (Yang et al., 2026). LIPUS also increases release of extracellular vesicles from bone marrow mesenchymal stem cells and enhances their anti-inflammatory effects, as validated in both *in vitro* experiments and allogeneic skin transplantation models (Li et al., 2023). In UV-induced skin aging mice, LIPUS enhanced recruitment of monocytes and macrophages and promoted clearance of senescent cells (Gwak et al., 2025). These inflammatory observations should not by themselves be interpreted as proof that NF-κB/MAPK signaling is required for the skin outcomes.

### 4.5 Signaling pathways involved in skin appendage regeneration

Regeneration of hair follicles and sweat glands involves developmental signals such as Wnt/β-catenin, Sonic hedgehog, fibroblast growth factor, and bone morphogenetic protein. These pathways play critical roles in normal hair follicle cycling and appendage formation. LIPUS-responsive piezoelectric membranes improved formation of skin appendage-like structures in a full-thickness skin defect model (Zhang Z. et al., 2025), and flexible piezoelectric patches promoted restoration of sweat gland structure and secretory function (Pi et al., 2024). These outcomes do not by themselves establish which of the developmental pathways listed above mediates the ultrasound response.

## 5 Ultrasound-regulated gene expression and epigenetics

Non-coding RNAs may mediate sustained regulation of ultrasound effects. In a rat acute wound model, diagnostic ultrasound combined with microbubbles improved re-epithelialization and collagen deposition, accompanied by downregulation of miR-26b-5p and altered GSK3β-related signaling (Huang et al., 2025). LIPUS treatment of stem cells alters the miRNA expression profile in their derived extracellular vesicles. Related studies have identified multiple differentially expressed miRNAs and predicted their target genes to be involved in cell cycle, MAPK, and Hippo signaling pathways (Yin et al., 2024). In human umbilical vein endothelial cells, LIPUS induces methylation changes at multiple CpG sites, with differential loci involving AKT pathway-related genes and concurrent pro-angiogenic effects (Li et al., 2022). Predicted miRNA targets and coincident methylation changes identify candidate mechanisms; they do not alone demonstrate that these pathways are required for ultrasound-mediated skin repair (Li et al., 2022; Yin et al., 2024).

## 6 Advances and translational challenges of ultrasound technology in skin repair and regeneration

### 6.1 Application of ultrasound in wound and scar therapy

Chronic wounds are often characterized by persistent inflammation, impaired blood perfusion, cellular senescence, and dysregulated extracellular matrix metabolism. A Cochrane review concluded that the quality of studies on venous leg ulcers is limited and results are inconsistent, and it remains uncertain whether therapeutic ultrasound provides additional healing benefits beyond standard compression therapy (Cullum and Liu, 2017). Most studies on diabetic foot ulcers have investigated ultrasound-assisted debridement rather than direct LIPUS stimulation of the wound bed.

A 2024 meta-analysis including 11 randomized controlled trials (696 patients) reported higher healing rates, but high heterogeneity was observed in healing time analysis, and no significant differences in wound perfusion or transcutaneous oxygen pressure were identified (Liu et al., 2024). A 40-patient randomized trial only demonstrated accelerated early healing, with no significant difference in 6-month healing rates (Lázaro-Martínez et al., 2020). Thus, current evidence is insufficient to determine its impact on amputation, hospitalization, or recurrence risk.

In animal models, however, therapeutic ultrasound improves fibroblast migration and promotes wound healing in diabetic and aged mice via the Ca²^+^/CaMKII/Tiam1/Rac1 pathway (Roper et al., 2015). The effects of 1 MHz pulsed ultrasound on granulation tissue formation and re-epithelialization in diabetic mice are sound pressure- and time-dependent, though individual responses vary (Vander Horst et al., 2021). Additionally, biofilms in chronic wounds sustain inflammation and reduce antimicrobial susceptibility (Mihai et al., 2024); mechanical perturbation and cavitation may disrupt biofilms or enhance drug penetration, but the incremental benefit relative to conventional debridement and antimicrobial therapy remains unclear. In a 20-patient clinical series, HIFU thermotherapy improved scar size, pigmentation, pain, and pruritus (Woźniak et al., 2025).

### 6.2 Advances in focused ultrasound for skin aging and tissue remodeling

Skin aging involves reduced and disorganized dermal collagen, abnormal elastic fibers, extracellular matrix degradation, and structural changes in subcutaneous soft tissues. Focused ultrasound creates localized thermal coagulation points at predetermined depths, inducing immediate collagen contraction and subsequent tissue remodeling. These changes primarily improve skin laxity and contour, representing tissue remodeling rather than complete structural and functional regeneration of the epidermis, dermis, and appendages.

Focused ultrasound shows efficacy in improving laxity and contour of the face, neck, and selected body regions (Haykal et al., 2025). Additionally, focused ultrasound modulates cilia in subcutaneous adipose tissue and adipose-derived stem cells (Oh et al., 2022), indicating that its penetration depth may extend below the dermis. Varying focusing frequencies and treatment depths result in different histological responses and patient perceptions (Hwang et al., 2025). One study combined topical formulations containing glutathione and hyaluronic acid with focused ultrasound to improve skin tone and hydration (Yi et al., 2024).

## 7 Ultrasound-mediated delivery and biomaterial strategies for skin repair and regeneration

### 7.1 Ultrasound-mediated transdermal delivery

The stratum corneum is the primary barrier limiting transdermal drug absorption. Macromolecular drugs, nucleic acids, proteins, and some nanocarriers generally cannot reach deep epidermal or dermal layers via passive diffusion. Ultrasound temporarily modifies stratum corneum structure or cell membrane permeability via physical effects such as mechanical vibration, acoustic streaming, and cavitation, thereby enhancing local drug delivery efficiency.

Based on the primary site of action, ultrasound-mediated delivery can be categorized into phonophoresis (targeting the stratum corneum barrier) and sonoporation (targeting cell membrane or vascular wall permeability). Low-frequency ultrasound phonophoresis mainly utilizes cavitation and the resulting microjets and shear stress to alter stratum corneum lipid arrangement and create transient hyperpermeable regions, facilitating drug diffusion from the skin surface into the epidermis and dermis (Gaikwad et al., 2024; Tao et al., 2025). While enhanced cavitation may increase delivery efficiency, it also elevates the risk of erythema, barrier damage, and local inflammation. The key goal of phonophoresis research is not to maximize permeability, but to achieve a balance between delivery efficiency and reversible barrier recovery.

Sonoporation refers to the transient increase in cell membrane or vascular wall permeability induced by ultrasound. This process can be induced by ultrasound alone or enhanced by exogenous microbubbles. When microbubbles undergo stable oscillation or rupture in an acoustic field, they generate local shear forces, microjets, and shock waves, enabling drugs, proteins, or nucleic acids to enter adjacent tissues and cells (Ho et al., 2021; Krut et al., 2022). Sonoporation exhibits certain spatiotemporal controllability, and pores typically close gradually after ultrasound cessation (Zhang et al., 2023); however, its safety still depends on peak negative pressure, exposure duration, and cavitation intensity.

### 7.2 Targeted delivery via ultrasound combined with microbubbles

Microbubbles typically consist of a gas core and a shell of lipid, protein, or polymer, and exhibit strong acoustic responsiveness (Rudakovskaya et al., 2022; Barmin et al., 2026). The advantage of ultrasound–microbubble combination therapy is that delivery timing and spatial range are controlled by the local acoustic field, and vascular permeability is enhanced to facilitate entry of circulating drugs or nanocarriers into target regions (Omata et al., 2021).

In diabetic wound models, ultrasound combined with microbubbles modulates macrophage polarization and inflammation-related signaling and promotes wound closure (Yang et al., 2026); additionally, in an acute rat wound model, ultrasound combined with microbubbles is associated with miR-26b-5p and GSK3β-related regulation, as well as improved re-epithelialization and collagen deposition (Huang et al., 2025). These findings suggest that microbubbles act not only as carriers, but also that their oscillation or rupture generates local mechanical effects that may contribute to wound outcome regulation.

### 7.3 Ultrasound-responsive hydrogels and local drug release

Hydrogels feature high water content, favorable tissue conformability, and tunable porous structure, making them suitable as wound dressings and local drug reservoirs. Stimuli-responsive hydrogels alter their network structure, swelling state, or degradation rate in response to external physical or chemical signals, enabling controlled drug release (Yeingst et al., 2022; Pan et al., 2023; Ren et al., 2024; Protsak and Morozov, 2025; Tian et al., 2025).

Therapeutic ultrasound can serve as an external trigger for local drug release from hydrogels. Ultrasound-responsive hydrogels for wound healing can be loaded with antimicrobial agents, anti-inflammatory factors, growth factors, oxygen-supplying materials, or extracellular vesicles. BaTiO_3_ piezocatalytic composite hydrogels generate reactive oxygen species under ultrasound triggering to exert local antibacterial effects while promoting wound healing in an infected full-thickness skin wound model (Liu et al., 2023). Polysaccharide-based smart hydrogels and stimuli-responsive wound dressings also show potential for maintaining a moist environment, absorbing exudate, and delivering active ingredients (Zubair et al., 2024; Ma T. et al., 2025; Fan et al., 2026).

### 7.4 Therapeutic ultrasound with nanofiber membranes, microneedles, and flexible patches

Nanofiber membranes possess a large specific surface area and a fibrous structure mimicking the extracellular matrix, providing support for cell adhesion, migration, and tissue ingrowth. Ultrasound can act as an external mechanical stimulus or trigger piezoelectric materials to generate local electrical signals.

LIPUS-responsive piezoelectric nanofiber membranes convert acoustic mechanical energy into local electrical stimulation, and promote wound healing, collagen remodeling, angiogenesis, and restoration of skin appendage-like structures in full-thickness skin defect models (Zhang Z. et al., 2025). A composite system consisting of a dual-layer chitosan-based conductive hydrogel and a piezoelectric film also shows potential for improving wound healing and reducing scar formation under ultrasound drive (Yao et al., 2025).

Flexible ultrasound or sono-piezoelectric patches conform to curved and irregular wound surfaces and provide relatively stable local stimulation over extended periods. Animal studies have found that flexible ultrasound patches accelerate chronic wound healing (Lyu et al., 2021), and sono-piezoelectric patches may promote sweat gland repair via remodeling of local nerves, blood vessels, and the cellular microenvironment (Pi et al., 2024).

Microneedles penetrate the stratum corneum to create microchannels, enhancing transdermal drug delivery efficiency. When combined with ultrasound, mechanical vibration, local acoustic streaming, or piezoelectric effects further promote drug diffusion. Piezoelectric-driven microneedle arrays have been used to enhance transdermal delivery of methotrexate and show therapeutic potential in psoriasis models (Li et al., 2025). This combined strategy reduces the difficulty of achieving penetration through an intact stratum corneum with ultrasound alone.

### 7.5 Ultrasound combined with growth factors, stem cells, and extracellular vesicles

Growth factors regulate migration, proliferation, and differentiation of keratinocytes, fibroblasts, and vascular endothelial cells. However, free growth factors in wounds are susceptible to proteolytic degradation or clearance by exudate. Ultrasound-mediated delivery and ultrasound-responsive carriers improve local retention of growth factors and enable controlled release timing via external triggering.

In acute and diabetic wound models, respectively, ultrasound combined with microbubbles is associated with altered expression of regeneration-related factors such as VEGF, and improved re-epithelialization and angiogenesis (Huang et al., 2025; Yang et al., 2026). Ultrasound-triggered oxygen release systems have also been investigated for diabetic foot ulcers, promoting wound healing by improving local oxygen supply (Zhuge et al., 2025).

Stem cells and their extracellular vesicles exert paracrine, immunomodulatory, and pro-angiogenic effects, but challenges remain regarding their retention, survival, and effective delivery in wounds. LIPUS enhances uptake of adipose-derived stem cell exosomes by target cells and augments their pro-angiogenic effects, thereby improving diabetic wound healing (Zhong et al., 2024). Enhancing local uptake of extracellular vesicles may represent a mechanism by which therapeutic ultrasound potentiates stem cell paracrine therapy.

Therapeutic ultrasound and oxygen-carrying microbubbles have also been applied to infected diabetic wounds. Carbon dot-loaded microbubbles provide an oxygen source under acoustic cavitation and enhance sonodynamic therapy, exhibiting both antibacterial and wound-healing effects in animal models (Ma G. et al., 2025) (**Figure 3**).

**Figure 3:**
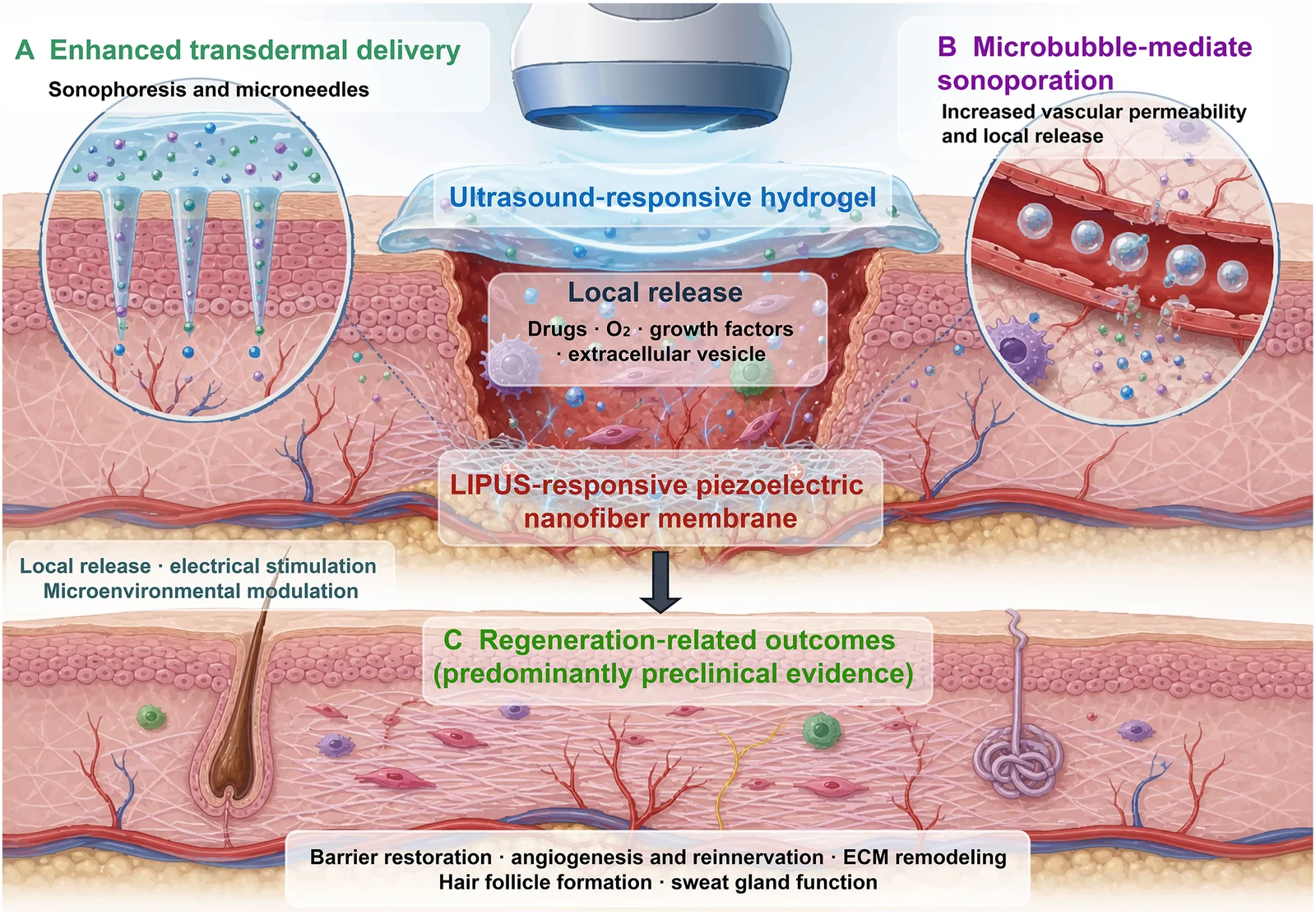
Ultrasound-mediated delivery and biomaterial-based strategies relevant to skin regeneration. **(A)** Low frequency sonophoresis, alone or in combination with microneedles, facilitates transport across the stratum corneum and enhances transdermal delivery. **(B)** Ultrasound-mediated microbubble oscillation and cavitation induce sonoporation, increase vascular permeability, and promote localized cargo release. Ultrasound-responsive hydrogels can provide controlled local delivery of drugs, oxygen, growth factors, and extracellular vesicles, whereas LIPUS-responsive piezoelectric nanofiber membranes convert acoustic mechanical energy into localized electrical cues. **(C)** These strategies have been associated with barrier restoration, angiogenesis, reinnervation, extracellular matrix remodeling, hair follicle formation, and recovery of sweat gland function. Most of these regeneration-related outcomes are currently supported by *in vitro* or preclinical animal studies, and their clinical efficacy and long-term safety require further investigation. LIPUS, low-intensity pulsed ultrasound.

## 8 Summary and perspectives

Collectively, therapeutic ultrasound participates in skin repair and regeneration via two main pathways: (1) modifying cellular and tissue behavior through mechanical stimulation or local thermal effects; and (2) serving as an external trigger to enhance local delivery or release of drugs, genes, extracellular vesicles, and biomaterials. LIPUS, low-frequency phonophoretic ultrasound, microbubble-assisted ultrasound, and focused ultrasound differ in their physical processes, therapeutic target layers, and safety margins.

Existing research has accumulated substantial evidence regarding fibroblast responses, re-epithelialization, macrophage regulation, angiogenesis, and matrix remodeling. Flexible patches, piezoelectric materials, and responsive carriers have expanded approaches for local stimulation and on-demand delivery.

At the mechanistic level, the integrin–focal adhesion–cytoskeleton axis, mechanosensitive ion channels, and calcium signaling provide a reasonable framework for understanding the mechanobiological effects of ultrasound. Signaling pathways such as MAPK/ERK, PI3K/Akt, TGF-β/Smad, and NF-κB may be involved in ultrasound-induced proliferation and inflammatory responses; however, the specific molecular mechanisms underlying therapeutic ultrasound regulation of skin tissue regeneration remain unclear.

In translational research, ultrasound has expanded avenues for local drug administration, antimicrobial therapy, extracellular vesicle delivery, and tissue engineering material stimulation. Nevertheless, most evidence remains at the *in vitro* or animal stage, and long-term safety evaluation of therapeutic ultrasound is critical for its clinical translation. For the biomaterial strategies in Section 6, translation additionally requires reproducible manufacture, sterilization and storage stability, and assessment of material degradation, particle or ion release, clearance and repeated-treatment toxicity (Luo et al., 2026). Piezoelectric output should be characterized in hydrated wound conditions; piezocatalytic and sonodynamic systems require balancing antibacterial reactive oxygen species generation against host-cell injury. Dressings, exudate and wound geometry may alter acoustic coupling, so tissue-interface exposure should be characterized alongside nominal device settings. Ultrasound-only, material-only and inactive-material controls are needed to distinguish acoustic, electrical, chemical and cargo effects. Human-relevant models and standard-care comparators should assess durable closure, recurrence and adverse effects; regenerative claims additionally require relevant functional outcomes, such as barrier recovery or appendage activity, rather than wound closure or collagen deposition alone.
